# Overexpression of *OsARD1* Improves Submergence, Drought, and Salt Tolerances of Seedling Through the Enhancement of Ethylene Synthesis in Rice

**DOI:** 10.3389/fpls.2019.01088

**Published:** 2019-09-10

**Authors:** Shanshan Liang, Wei Xiong, Cuicui Yin, Xiaodong Xie, Ya-jun Jin, Siju Zhang, Bo Yang, Guoyou Ye, Shouyi Chen, Wei-jiang Luan

**Affiliations:** ^1^College of Life Sciences, Tianjin Key Laboratory of Animal and Plant Resistance, Tianjin Normal University, Tianjin, China; ^2^State Key Lab of Plant Genomics, Institute of Genetics and Developmental Biology, Chinese Academy of Sciences, Beijing, China; ^3^College of Agriculture, Resources and Environmental Sciences, Tianjin Agricultural University, Tianjin, China; ^4^Genetics and Biotechnology Division, International Rice Research Institute (IRRI), Los Baños, Philippines

**Keywords:** *OsARD1*, ethylene, rice, submergence tolerance, drought tolerance, salt tolerance

## Abstract

Acireductone dioxygenase (ARD) is a metal-binding metalloenzyme and involved in the methionine salvage pathway. In rice, *OsARD1* binds Fe^2+^ and catalyzes the formation of 2-keto-4-methylthiobutyrate (KMTB) to produce methionine, which is an initial substrate in ethylene synthesis pathway. Here, we report that overexpression of *OsARD1* elevates the endogenous ethylene release rate, enhances the tolerance to submergence stress, and reduces the sensitivity to drought, salt, and osmotic stresses in rice. *OsARD1* is strongly induced by submergence, drought, salinity, PEG6000, and mechanical damage stresses and exhibits high expression level in senescent leaves. Transgenic plants overexpressing *OsARD1* (OsARD1-OE) display fast elongation growth to escape submergence stress. The ethylene content is significantly maximized in OsARD1-OE plants compared with the wide type. OsARD1-OE plants display increased shoot elongation and inhibition of root elongation under the submergence stress and grow in dark due to increase of ethylene. The elongation of coleoptile under anaerobic germination is also significantly promoted in OsARD1-OE lines due to the increase of ethylene content. The sensitivity to drought and salt stresses is reduced in OsARD1-OE transgenic lines. Water holding capacity is enhanced, and the stomata and trichomes on leaves increase in OsARD1-OE lines. Drought and salt tolerance and ethylene synthesis–related genes are upregulated in OsARD1-OE plants. Subcellular localization shows that *OsARD1* displays strong localization signal in cell nucleus, suggesting *OsARD1* may interact with the transcription factors. Taken together, the results provide the understanding of the function of *OsARD1* in ethylene synthesis and abiotic stress response in rice.

## Introduction

Acireductone dioxygenase (ARD) is a metal-binding protein family belonging to the cupin superfamily (Dunwell et al., 2004). The methionine salvage pathway is a ubiquitous pathway found in plants, animals, and bacteria. ARD catalyzes the penultimate step in the methionine salvage pathway, the oxidative decomposition of substrate acireductone (1,2-dihydoxy-3-keto-5-[thiomethyl]-pent-1-ene) to formate and 2-keto-4-(thiomethyl) butyrate (KMTB), the keto-acid precursor of methionine. In the bacterium *Klebsiella oxytoca*, ARD has dual activity depending on the binding metal ion cofactor. ARD bound with Fe^2+^ catalyzes on-pathway chemistry leading to production of formate and the keto-acid precursor to methionine, whereas ARD bound with Ni^2+^ catalyzes an off-pathway shunt leading to production of formate, carbon monoxide, and methylthiopropionate (MTP) (Wray and Abeles, 1995; Dai et al., 1999). In mouse (*Mus musculus*), ARD bound with Fe^2+^ catalyzes on-pathway chemistry resulting in formate and the ketoacid precursor of methionine, whereas the Ni^2+^, Co^2+^, or Mn^2+^ forms catalyze off-pathway chemistry, similar as ARD from *Klebsiella* (Deshpande et al., 2016). In *Arabidopsis*, *ARD1* encodes an active metalloenzyme and interacts with AGB1, a heterotrimeric G protein β (Gβ), to contribute to the production of ethylene and control hypocotyl length by modulating cell division (Friedman and Wang, 2011). In rice, *OsARD1* is strongly induced by ethylene and catalyzes the formation of KMTB to further produce methionine in the methionine salvage pathway (Sauter et al., 2005).

Ethylene has been extensively studied and found to play diverse functions in cell elongation, plant senescence, fruit ripening, and plant growth and development. However, the function and mechanism of ethylene in stress tolerance is unclear and rarely reported. It has been well elucidated that ethylene is a major regulator of submergence tolerance in rice (Xu et al., 2006). *Submergence 1* (*Sub1*) locus in rice contains a cluster of three genes including *Sub1A*, *Sub1B*, and *Sub1C*. *Sub1B* and *Sub1C* are invariably present in all rice accessions, whereas *Sub1A* is present only in submergence tolerance-specific rice accessions (Xu et al., 2006). *Sub1A* encodes putative ethylene response factors (ERF) and is responsible for submergence tolerance. Overexpression of *Sub1A* enhanced tolerance to submergence stress in submergence-intolerant rice (Xu et al., 2006). The study of the molecular mechanism of deepwater response indicated that *SNORKEL1* and *SNORKEL2* were induced and then triggered internode elongation *via* gibberellic acid (GA) hormone due to the accumulation of ethylene in rice plants under deepwater condition (Hattori et al., 2009). Two antithetical models were proposed to explain how plants overcome submergence stress under deepwater or submergence conditions. Quiescence strategy could make the plants avoid any unnecessary energy consumption by limiting underwater growth and conservation of energy and carbohydrates (Bailey-Serres and Voesenek, 2008). The antithetical model is the low oxygen escape strategy. Some species can stimulate the elongation growth of petioles, stems, or leaves through the ethylene and GA-promoted fast elongation to restore contact between leaves and atmosphere before the depletion of energy (Bailey-Serres and Voesenek, 2008; Fukao and Bailey-Serres, 2008).

Studies reveal that several *ERF* genes in plants play important roles in drought and salt stresses. *SodERF3* encodes a DNA-binding protein to serve as a transcriptional regulator of ethylene-responsive factor in sugarcane (*Saccharum officinarum*). Ectopic expression of *SodERF3* in tobacco (*Nicotiana tabacum*) enhances salt and drought tolerance (Trujillo et al., 2008). *OsTSRF1* is an ERF transcription factor and improves osmotic and drought tolerance by modulating the expression of stress-responsive gene such as *MYB*, *MYC*, and proline synthesis and photosynthesis-related genes in rice (Quan et al., 2010). *OsERF3* modulates drought tolerance by interacting with *OsDERF1* to negatively regulate ethylene production (Zhang et al., 2013). *OsERF71* is strongly induced by drought and salinity and enhances drought tolerance through altering root structure by the elevation of cell wall loosening and lignin biosynthetic genes in rice (Lee et al., 2016).

In addition, several genes related to the ethylene signal transduction pathway have been reported to play important roles in resistance to drought and salt stresses. *NTHK1*, a type II ethylene receptor homolog gene, encodes a histidine kinase in tobacco to play an important role in salt stress responses (Cao et al., 2007). Overexpression of *NTHK1* enhances tolerance to salt in tobacco and *Arabidopsis*. EIN2, a central membrane protein of ethylene signaling, can interact with MA3 domain-containing protein ECIP1 to regulate ethylene response and salt tolerance in *Arabidopsis* (Lei et al., 2011). Ethylene overproducer 1-like gene (*OsETOL1*), a homolog of *Arabidopsis* *ETO1*, encodes a putative E3 ubiquitin ligase and modulates drought tolerance by interacting with *OsACS2* to regulate negatively ethylene biosynthesis in rice (Du et al., 2014). *MHZ6/OsEIL1* and *OsEIL2*, two rice transcriptional regulators of ethylene signaling, negatively regulate salt tolerance in rice. Knockout of *MHZ6/OsEIL1* or *OsEIL2* improves salt tolerance, whereas the overexpression of *MHZ6/OsEIL1* or *OsEIL2* enhances salt hypersensitivity (Yang et al., 2015).

Although ARD was confirmed to be an active enzyme in the methionine salvage pathway, the biological function of *OsARD1* remains unclear in plants. To understand the biological function of *OsARD1* in rice, we generated transgenic lines overexpressing *OsARD1* and tested their performance under various abiotic stresses. Our results show that *OsARD1* is induced by submergence, drought, salinity, PEG6000, and mechanical damage. Transgenic lines overexpressing *OsARD1* have elevated endogenous ethylene release rate, improved water holding capacity, and reduced sensitivity to submergence, drought, and salt stress, suggesting that *OsARD1* plays an important role in abiotic stresses tolerance in rice.

## Materials and Methods

### Plant Materials

The rice variety Zhonghua11 (*Oryza sativa* L. ssp. *japonica*) and transgenic plants overexpressing *OsARD1* were used in this study. Transgenic plants were generated from Zhonghua11 using the *Agrobacterium*-mediated transformation method described by Hiei et al. (1994). Plants were planted in the experimental field of Tianjin Normal University, Tianjin and Lingshui, Hainan Province, China.

### Construction of the Overexpression Vector and Rice Transformation

*OsARD1* cDNA with full-length open reading frame was amplified from total RNA of 30-day-old seedlings by RT-PCR by specific primers: OARD1-F: 5’-TTAggtaccTTCCACCCCGCAATCCACAT-3’ and OARD1-R: 5’-GTTgtcgacGTGCAGGAGCCCAACAAAAC-3.’ The bases in lowercase are recognition sites for restriction endonuclease enzymes *Kpn*I and *Sal*I. The resulting fragment was cloned into an empty pCAMBIA2300 binary vector with double CaMV 35S promoters to obtain the recombinant vector. After validation by sequencing, the resultant vector was introduced into WT Zhonghua11 using *Agrobacterium*-mediated transformation method to generate transgenic plants overexpressing *OsARD1*.

### GUS Staining Assay

A fragment of 2,169-bp upstream of the *OsARD1* putative transcription start codon was amplified from genomic DNA of Zhonghua11 with specific primers ARD1-GUSF: 5’-CTAaagcttGTTGCTTGCGTGCCATTTAT-3’ and ARD1-GUSR: 5’-TCAgaattcTTCGTTCTCCATGTGGATTG-3.’ The bases in lowercase are restriction endonuclease recognition sites. The amplified DNA fragment was inserted into a pCAMBIA1391Z empty vector to fuse with the *GUS* reporter gene to produce the recombinant vector *pOsARD1*::*GUS*. After sequencing validation, the recombinant vector was introduced into WT Zhonghua11 *via Agrobacterium*-mediated transformation. Different transgenic rice tissues and organs were collected and incubated in GUS staining solution (100 mmol L^−1^ phosphate buffer [pH 7.0], 10 mmol L^−1^ EDTA, 1 mmol L^−1^ potassium ferricyanide, 0.1% Triton X-100, and 2 mmol L^−1^ X-Gluc) overnight at 37°C. Samples were then washed with 70% ethanol and fixed in FAA (5% formaldehyde, 5% acetic acid, and 80% ethanol) for microscopic observation.

### Subcellular Localization of OsARD1

The full-length coding sequence of *OsARD1* without stop codon was amplified from the cDNA of 60-day-old rice leaf by RT-PCR using specific primers: ARD1-GFP-F: 5’-GTTgagctcATGGAGAACGAATTCCAGGA-vw3’ and ARD1-GFP-R: 5’-TCTgtcgacGAAGCCTTCAACTGCTTGAT-3.’ The amplified cDNA fragment was cloned into a pCAMBIA35S-GFP empty vector and fused in-frame with green fluorescent protein (GFP) to produce the resultant vector *p35S::OsARD1-GFP*. After validation by sequencing, the fused vector and empty vector *p35S::GFP* were introduced into the *Agrobacterium tumefaciens* strain and then transiently transformed into tobacco epidermal cell by injecting tobacco (*N. tabacum*) leaves. After culturing for 2 days at 25°C, the tobacco epidermis was visualized under a fluorescence microscope (Leica, DM5000B, Germany). The fusion vector *p35S::OsARD1-GFP* and empty vector *p35S::GFP* were also transformed into onion (*Allium cepa*) epidermal cells by gene bombardment (the Bio-Rad PDS-1000/He device, USA). Bombarded epidermal cells were incubated for 2 days at 25°C in the dark. The epidermal cell layers were then observed using a fluorescence microscope (Leica, DM5000B, Germany).

### Quantitative RT-PCR (qRT-PCR) Analysis

For analysis of gene expression in transgenic lines, total RNAs were isolated from 65-day-old leaves using TRIzol reagent (Invitrogen, USA) and treated with DNase I (NEB, USA). For the expression of stress-related genes and ethylene synthesis–related genes, RNAs were isolated from the 70-day-old leaves of OsARD1-OE and WT lines, respectively. cDNA was synthesized from 1 μg of total RNA using HiScript II Reverse Transcriptase (Vazyme, Nanjing, China). RT-PCR analysis was performed with 1 µl cDNA and gene-specific primers. qRT-PCR was performed using SYBR Green PCR mix (Vazyme, Nanjing, China) in 7500 Fast Real-time PCR System (ABI, USA) with three replicates. The 2^−ΔΔCt^ method (Livak and Schmittgen, 2001) was used for the analysis of relative gene expression. *OsActin1* was used as an internal control for the relative quantification of target gene expression. All primers used in this research are listed in the **Supplementary Table S1**.

### Expression Analysis of *OsARD1* After Phytohormone and Abiotic Stress Treatment

Zhonghua11 seedlings were cultivated in rice nutrient solution (Yoshida et al., 1972) and grown in plant growth chamber (MMM, ClimaCell, Germany) at 28°C, 14 h light and 10 h dark. Fourteen-day-old seedlings were translocated into nutrient solution containing 20% PEG6000, 200 mM NaCl, or mechanical damage seedlings by tweezers. Total RNAs were isolated from the seedlings using TRIzol solution (Invitrogen, USA). qRT-PCR was used to analyze the expression of *OsARD1*. For phytohormone treatments, Zhonghua11 seedlings were planted in soil pot in plant growth chamber at 28°C, 14 h light and 10 h dark. Forty-day-old seedlings were subject to phytohormone treatments by spraying with 0.1 mM ethephon, 0.1 mM IAA, 0.1 mM ABA, and 0.1 mM MeJA solution, respectively. Water was sprayed as control. Leaves from treated seedlings were collected for total RNA isolation to analyze *OsARD1* expression at 0, 1, 3, 6, 12, and 24 h, respectively, using the same method described as above.

### Assay of Ethylene Release Rate in OsARD1-OE Lines

The determination of ethylene release rate is performed according to the procedure described by Yang et al. (2017). The seeds of four OsARD1-OE lines (OE-20, OE-35, OE-61, and OE-63) and WT were soaked in tap water for 2 days and incubated for 1 day at 28°C for germination. The pre-germinated seeds were put on absorbent filter paper in the uncapped airtight plastic bottle (max volume 500 ml) and grew for 7 days without lid. For each independent line, three replicates were set up in different bottles. Seven days later, the bottles were sealed with lid. The ethylene released by each sample was obtained after preservation of 17 h in the dark and was assayed using a GC2014 Gas Chromatographer (Shimadzu, Tokyo, Japan).

### Phenotyping for Anaerobic Germination

Seeds of four OsARD1-OE lines (OE-20, OE-35, OE-61, and OE-63) and WT were surface sterilized in 20% diluted bleach (6–7% NaClO) for 20 min and then thoroughly rinsed with sterilized water. For control samples, 15 sterilized seeds were placed on water-soaked filter tissues in capped 20-cm tall glass tubes. For submerged samples, the sterilized seeds were submerged in sterilized water (up to 5 cm in the 20-cm tube). The germination experiment was conducted at room temperature with a 16-h light/8-h dark cycle. After 14 days, coleoptile length was measured using a standard ruler, and the anaerobic response index was calculated by following the formula: The ARI = coleoptile length under submergence condition (the treatment group)—coleoptile length under normal condition (the control group) (Hsu and Tung, 2015).

### Treatment of Transgenic Lines With Submergence Stress and Dark

Pre-germinated OsARD1-OE and WT seeds that were sowed in plastic pot filled with soil were completely submerged under fresh water 35 cm deep. The time of leaf growing from under water to air was observed and recorded. For dark treatment, WT and OsARD1-OE lines were planted in the dark in the climate chamber (28°C). The length of shoot and root were measured. For *OsARD1* and submergence-related genes expression analysis of submergence response, 10-day-old seedlings of WT and OsARD1-OE lines were submerged with water depth of 35 cm. After 7 days of submergence treatment, the seedlings were out of water to desubmergence for 3 days. Total RNAs were isolated from seedlings at different time of submergence and desubmergence. qRT-PCR was performed using SYBR Green PCR Master Mix Kit (Vazyme, Nanjing, China) in 7500 Fast Real-time PCR System (ABI, USA) to analyze the expression of submergence-related genes in OsARD1-OE and WT plants.

### Scanning Electron Microscope Observation of Leaf Surface, Water Holding Capacity, and Water Content Measurement in the Leaves

Five flag leaves of 65-day-old were first removed from main stem of WT and OsARD1-OE plants in the field, respectively. A leaf disk (area around 0.3 cm^2^) was then removed by hole punch from the center of the leaf blade of transgenic lines and WT. Excised leaf disks were observed for the trichome and stomata density on the adaxial surface of leaf directly under the scanning electron microscope (Hitachi TM3030, Japan). Three images of fields of vision under the microscope were taken digitally and used to count the number of trichome and stomata. The trichome or stoma density was calculated as the trichome or stoma number per disk divided by the disk area and averaged among the five leaf blades.

For water holding capacity test, leaves at different leaf age including the penultimate, antepenultimate, and the fourth leaves from the bottom from main stem of WT and OsARD1-OE lines were excised and exposed in the open air. The time when the leaves displayed dehydration phenotype was recorded.

Relative water content (RWC) and WC were measured at heading stage. The penultimate leaves from three different plants were sampled at random. The fresh weight (FW) was immediately recorded after leaf excision. The leaves were left in distilled water for 24 h, and the turgid weight (TW) was recorded. The dry weight (DW) was then measured after 48 h at 60°C. The RWC was calculated as RWC (%) = [(FW–DW)/(TW–DW)] × 100%. The WC was calculated as WC (%) = (FW–DW)/FW ×100% (Barrs and Watherley, 1968). All samples were prepared in three replicates. The mean value of each line was calculated, and the statistical significance was determined using the Student’s t-test.

### Phenotypic Analysis of Transgenic Plants Under Drought and Salinity Conditions

Two independent overexpression transgenic lines (OsARD1-OE-35 and 63) were planted in the pots for drought treatment. Two-week-old seedlings were subject to drought stress by stopping water. For PEG and salt treatments on agar medium, OsARD1-OE and WT were planted on agar medium containing 5% PEG6000 or 50, 100, and 150 mM NaCl, respectively. For PEG and salt treatments in nutrient solution, OsARD1-OE and WT were cultured in rice nutrient solution (Yoshida et al., 1972). For each experiment, a control group was set up. Fourteen-day-old seedlings were transferred to the nutrient solution with 20% PEG6000 or 200 mM NaCl, respectively. After treatments, the phenotypic traits were observed and recorded. The length of shoot and root, death rate of high-concentration salinity group were measured or calculated.

## Results

### Expression Profile of *OsARD1*

**Figure 1:**
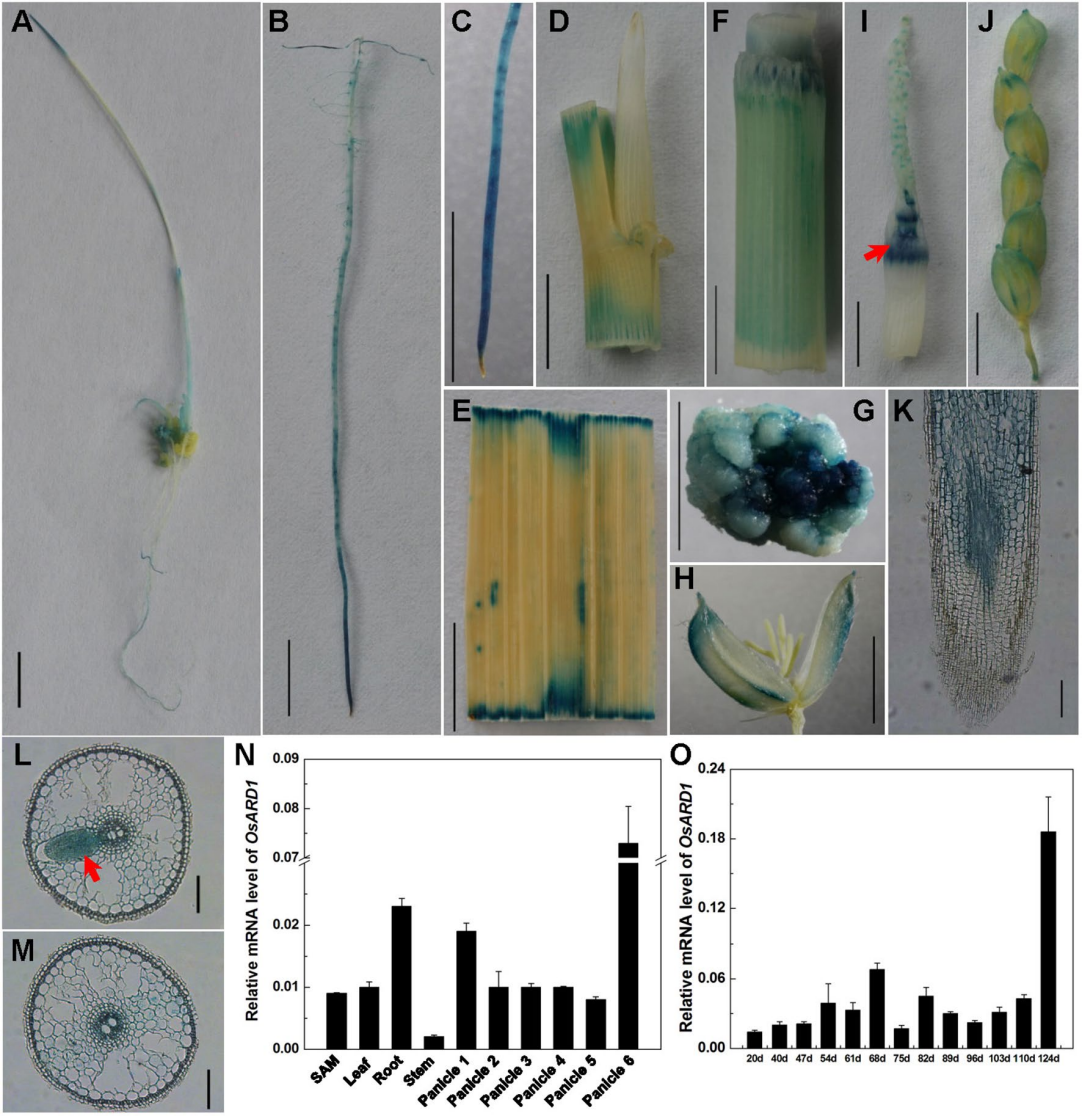
Expression patterns of *OsARD1*. **(A**–**J)** GUS staining in different tissues and organs. **(A)** Seedling in tissue culture. **(B)** Root. **(C)** Root tip. **(D)** Leaf sheath with ligule and auricle. **(E)** Leaf blade. **(F)** Stem. **(G)** Callus. **(H)** Floral. **(I)** 2-cm-long young panicle, arrowhead is intercalary meristem. **(J)** 22-cm-long panicle. **(K**–**M)** The section of *GUS* expression in root tip. **(K)** Longitudinal section. **(L**–**M)** Cross section, arrowhead is meristematic cell of lateral-root primordial. **(N)** qRT-PCR analysis of *OsARD1* expression in different organs. Panicle 1: 0.3–0.5-cm young panicles, panicle 2: 1–2-cm young panicles, panicle 3: 3–4-cm young panicles, panicle 4: 5–6-cm young panicles, panicle 5: 10–11-cm young panicles, panicle 6: panicle at heading stage. **(O)** qRT-PCR analysis of *OsARD1* expression at different developmental stages. *OsActin1* was used as an internal control for the relative quantification of target gene expression in qRT-PCR. Scale bars: 0.5 cm for **A**–**J** and 100 μm for **K**–**M**.

To reveal the biological function of *OsARD1* in rice, we analyzed the expression pattern of *OsARD1* in different organs and tissues. A fusion vector of the *OsARD1* promoter region and *GUS* reporter gene was constructed and introduced into Zhonghua11 to generate transgenic plants. GUS staining indicated that *OsARD1* exhibited different expression level in different organs and tissues of transgenic rice. Strong expression was detected in root (**Figure 1B**), callus (**Figure 1G**), intercalary meristem (shown by arrowhead in **Figure 1I**), and green palea and lemma of the floral organ (**Figures 1H–J**), especially in root tip (**Figure 1C**). We further examined the activity of GUS driven by the *OsARD1* promoter in root using histological section method and found that GUS staining was observed mainly in meristematic zones and elongation zone, but no staining in root cap cell (**Figure 1K**). Moreover, GUS staining was observed mainly in vascular bundle and parenchymatous cell of roots (**Figures 1L, M**), especially displaying high expression in meristematic cell of lateral-root primordial (**Figure 1L** indicated with arrowhead). GUS staining was also observed in sheath, leaf, stem, callus, and young panicle in transgenic plants (**Figures 1A, D–G and I**). In floral organs, GUS staining was mainly detected in palea and lemma, but not in stamens and pistil (**Figures 1H–J**).

We isolated RNA from organs of SAM, young roots, young leaf, stem of the second internode, and different stages of panicles to analyze the expression of *OsARD1*. qRT-PCR showed that *OsARD1* expressed in all these organs, with higher expression level in the heading panicle and young root, moderate expression in SAM, young leaf and younger panicle (panicles 1–5), and low expression level in stem (**Figure 1N**). This result was consistent with the GUS staining result. In addition, we analyzed *OsARD1* expression profile of penultimate leaf at different developmental stages in the field and found that the *OsARD1* mRNA level was comparable with each other in mature and green leaf (from 54 to 110 days), but slightly lower at seedling stages (from 20 to 47 days) (**Figure 1O**). Interestingly, *OsARD1* expression was strongly induced in senescent leaves in yellow color at mature stage (124 days) (**Figure 1O**), suggesting that *OsARD1* expression could be induced at senescent stage of leaf.

### Subcellular Localization of OsARD1

**Figure 2:**
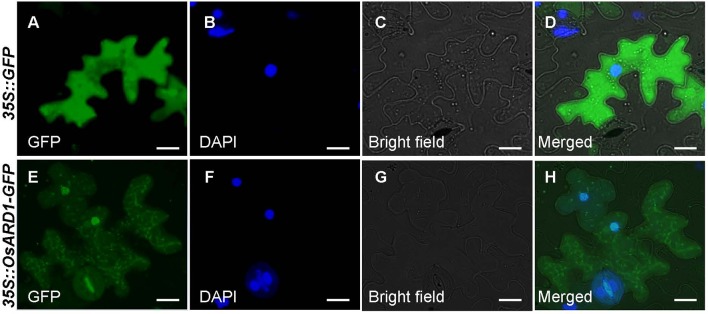
Subcellular localization of OsARD1. **(A**–**D)** The transient expression of *pCAMBIA35S::GFP* empty vector in tobacco epidermal cells. **(E**–**H)** The transient expression of *pCAMBIA35S::OsARD1-GFP* fusion vector in tobacco epidermal cells. Scale bars: 25 μm.

A fused vector containing *OsARD1* and *GFP* was constructed to investigate the subcellular localization of *OsARD1*. After sequencing validation, *pCAMBIA35S::OsARD1-GFP* vector and empty vector *pCAMBIA35S::GFP* were introduced into tobacco leaves to produce transient expression *via* infiltration of *A. tumefaciens* strain EHA105. We observed that OsARD1-GFP signal was located in the tobacco cell nucleus and cytoplasm, with stronger signal in cell nucleus than in cell cytoplasm (**Figures 2E–H**). In contrast, the GFP signal generated by the empty GFP vector was detected throughout the cell (**Figures 2A–D**). Meanwhile, the fused vector and empty vector were transformed into endepidermis cell of onion using gene gun. The result showed that OsARD1-GFP signal appeared in the cell nucleus and cytoplasm (**Figures S1D–F**), with stronger signal in the cell nucleus. The GFP signal of the empty vector was detected throughout the cell (**Figures S1A–C**). The results from the two transient expression methods were consistent, indicating that OsARD1 localized in the cell nucleus and cytoplasm.

### Expression Profile of *OsARD1* Under Various Abiotic Stresses and Phytohormone

**Figure 3:**
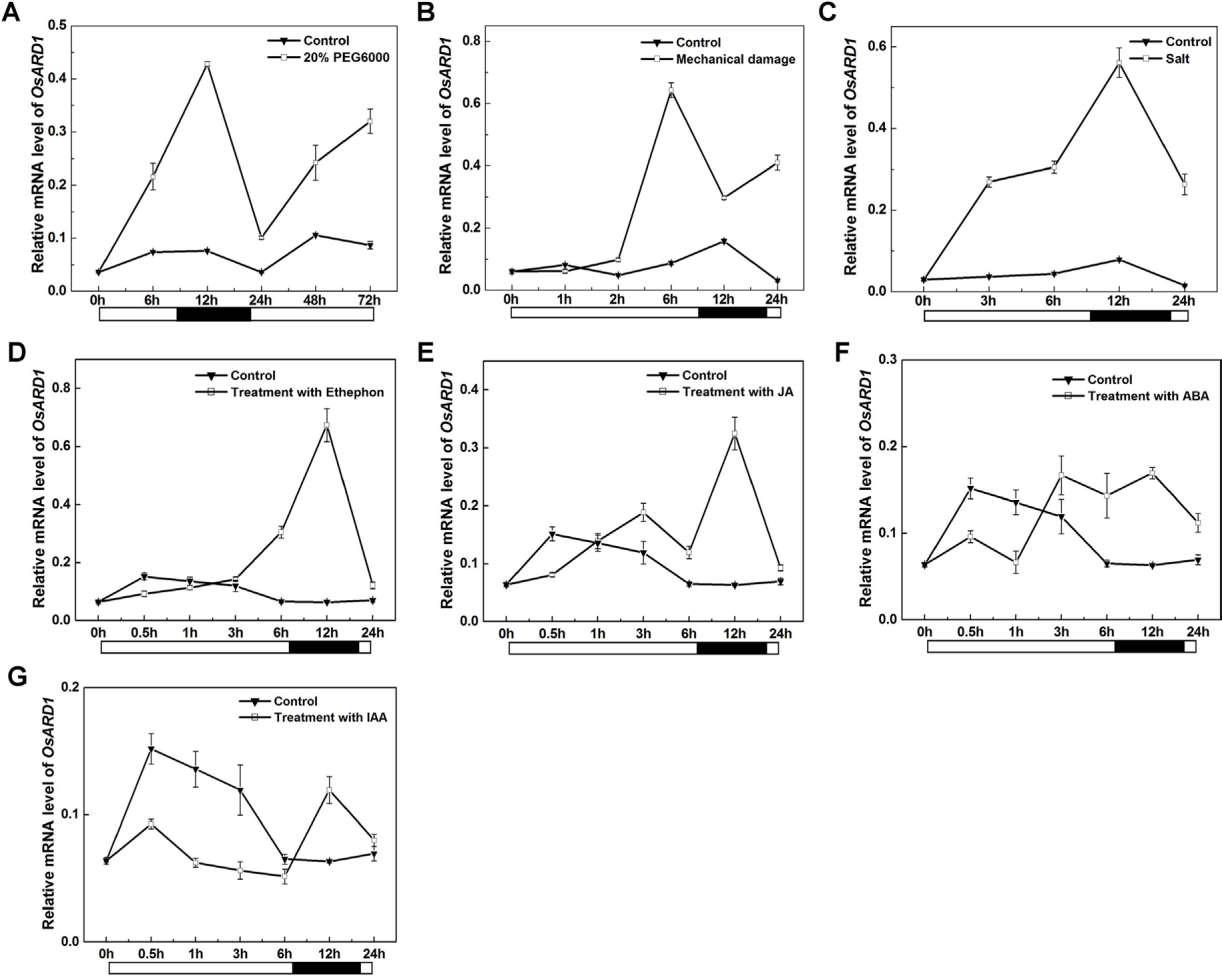
Expression analysis of OsARD1 induced by various abiotic stresses and phytohormone treatments. **(A**–**C)** qRT-PCR analysis of *OsARD1* expression in different abiotic stresses. **(D**–**G)** qRT-PCR analysis of *OsARD1* expression after treatment with ethephon, MeJA, ABA, and IAA. About 30-day-old WT seedlings were subject to PEG6000, mechanical damage and salt stresses and sprayed with ethephon, MeJA, ABA, and IAA, respectively. Water treatment was used as a control for phytohormone treatments. RNAs were isolated from leaves to analyze the expression of *OsARD1* by qRT-PCR. Black solid bars indicate the dark stage, and open bars indicate the light stage.

Our result indicated that mRNA level of *OsARD1* was strongly induced in senescent leaves. The senescent process of plant is usually associated with environmental stress. It is natural to speculate that *OsARD1* may be induced by different abiotic stresses. Therefore, we examined *OsARD1* expression level under different abiotic stresses by qRT-PCR. The results showed that *OsARD1* was strongly and rapidly induced by PEG6000, mechanical damage, and high-concentration salt (**Figures 3A–C**). In 20% PEG6000 and high-concentration salt treatments, *OsARD1* expression was induced within 6 h and reached to the peak at 12 h (**Figures 3A, C**). In the mechanical damage treatment, *OsARD1* expression peaked at 6 h after treatment (**Figure 3B**).

Given the close relationship between phytohormone and abiotic stresses, we further analyzed *OsARD1* expression in different phytohormone treatments including ethylene, MeJA, ABA, and IAA. The result showed that *OsARD1* was strongly induced by ethylene and MeJA (**Figures 3D, E**) and slightly induced by ABA (**Figure 3F**). Whereas *OsARD1* expression was inhibited by IAA (**Figure 3G**), suggesting that IAA might have a suppressed effect on the expression of *OsARD1*.

### Overexpression of *OsARD1* Elevated Endogenous Ethylene Release Rate

**Figure 4:**
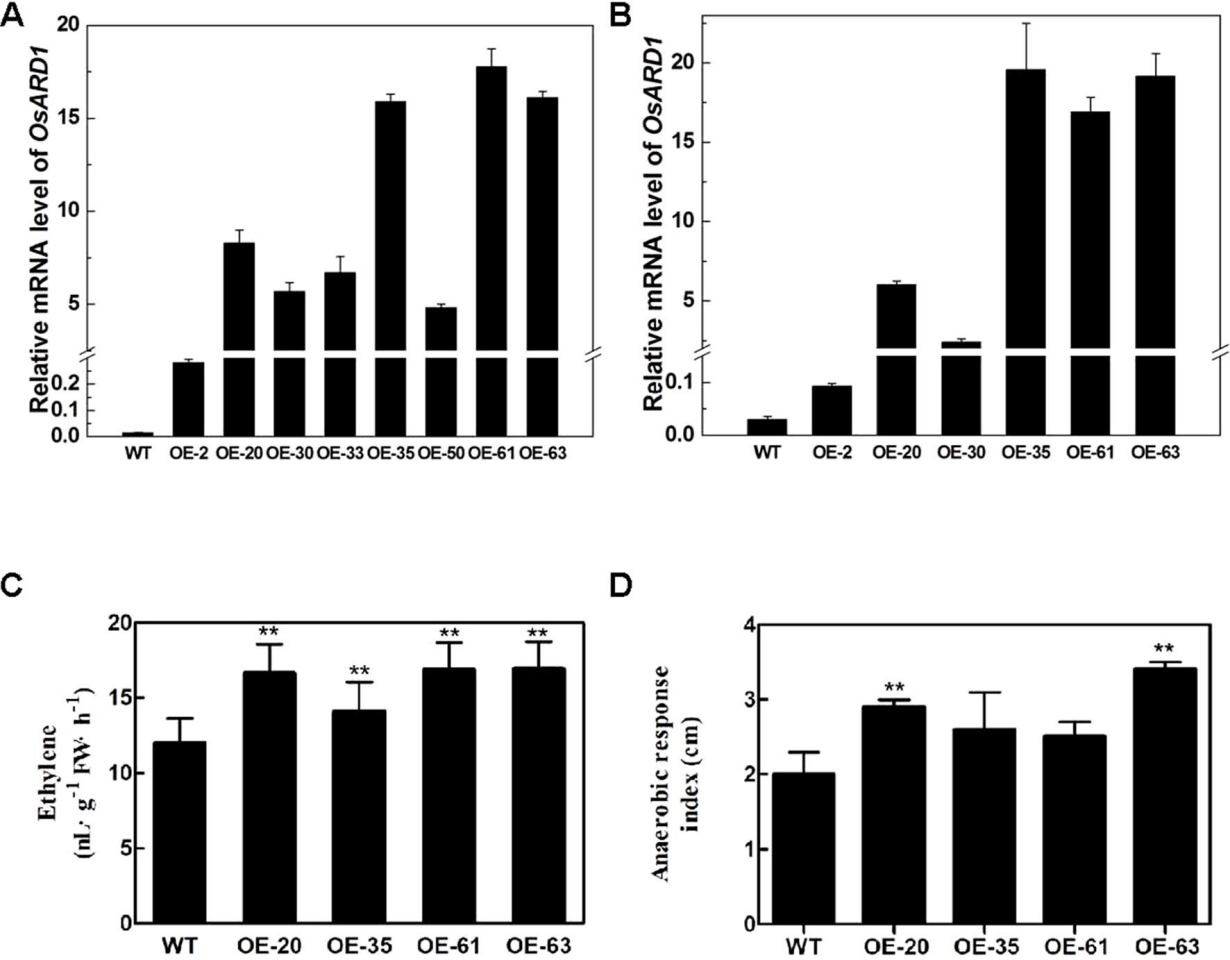
The endogenous ethylene release rate and anaerobic response index in transgenic lines. **(A**–**B)** *OsARD1* expression level in transgenic lines at T_0_ **(A)** and T_1_ generations **(B)**. **(C)** Endogenous ethylene release rate of OsARD1-OE lines and WT. ** denotes t-test at 0.01 significance probability level (n = 13–16). **(D)** Anaerobic response index of OsARD1-OE lines after 14 days under anaerobic germination.

To elucidate the function of *OsARD1* in rice, we constructed an overexpression vector of *OsARD1* and introduced it into Zhonghua11 to obtain transgenic plants. We totally produced 65 T_0_ transgenic plants from 11 independent lines. *OsARD1* expression level was examined by qRT-PCR in randomly selected T_0_ transgenic plants. The result showed that *OsARD1* mRNA level was significantly elevated in the detected eight independent transgenic lines, with the highest line rising thousandfold compared to WT plants (**Figure 4A**). No obvious phenotypic change was observed in T_0_ plants in the field. Six T_0_ generation transgenic lines were advanced to produce T_1_ generation lines. Expression analysis of T_1_ generation plants indicated that *OsARD1* expression level was strongly enhanced in different overexpression lines, suggesting that the pattern of overexpression was stably heritable (**Figure 4B**).

Since *OsARD1* encodes an active ARD which is indirectly associated with ethylene synthesis pathway, we measured the ethylene production in OsARD1-OE lines and WT, respectively. The four tested transgenic lines had significantly higher endogenous ethylene release rate than WT plants (**Figure 4C**), with increase percentage ranging from 17.7 to 41.3%. The ethylene release rate in the highest line OE-63 increased by 41.3% compared to WT, followed by OE-20 and OE-61 which increased by 40.8 and 39.0%, respectively. OE-35 had the lowest increase percentage, which was 17.7% (**Figure 4C**). The results indicated that overexpressing *OsARD1* enhanced the endogenous ethylene production in rice, suggesting that *OsARD1* plays an important role in ethylene synthesis pathway.

### Overexpression of *OsARD1* Promoted the Elongation of Coleoptile Under Anaerobic Germination

The elongation of coleoptile under anaerobic germination will be speeded up due to the increase of ethylene production. We further carried out the experiment of anaerobic germination using OsARD1-OE lines and WT. For the control group, there was no difference in the coleoptile length between the four tested transgenic lines and WT. However, the coleoptile lengths of the four tested transgenic lines were longer than WT under anoxia. The difference in coleoptile length between control plants and those submerged in water was designated the ARI, which represented the level of tolerance to anaerobic germination. The ARI of four transgenic lines was all higher than that in WT (**Figure 4D**). ARI of two transgenic lines (OE-63 and OE-20) increased approximately by up to 50% compared with WT. Those results indicated that overexpression of *OsARD1* promoted the elongation of coleoptile under anaerobic germination in OsARD1-OE lines due to the increase of ethylene production.

### Overexpression of *OsARD1* Enhances Submergence Tolerance at Seedling Stage

**Figure 5:**
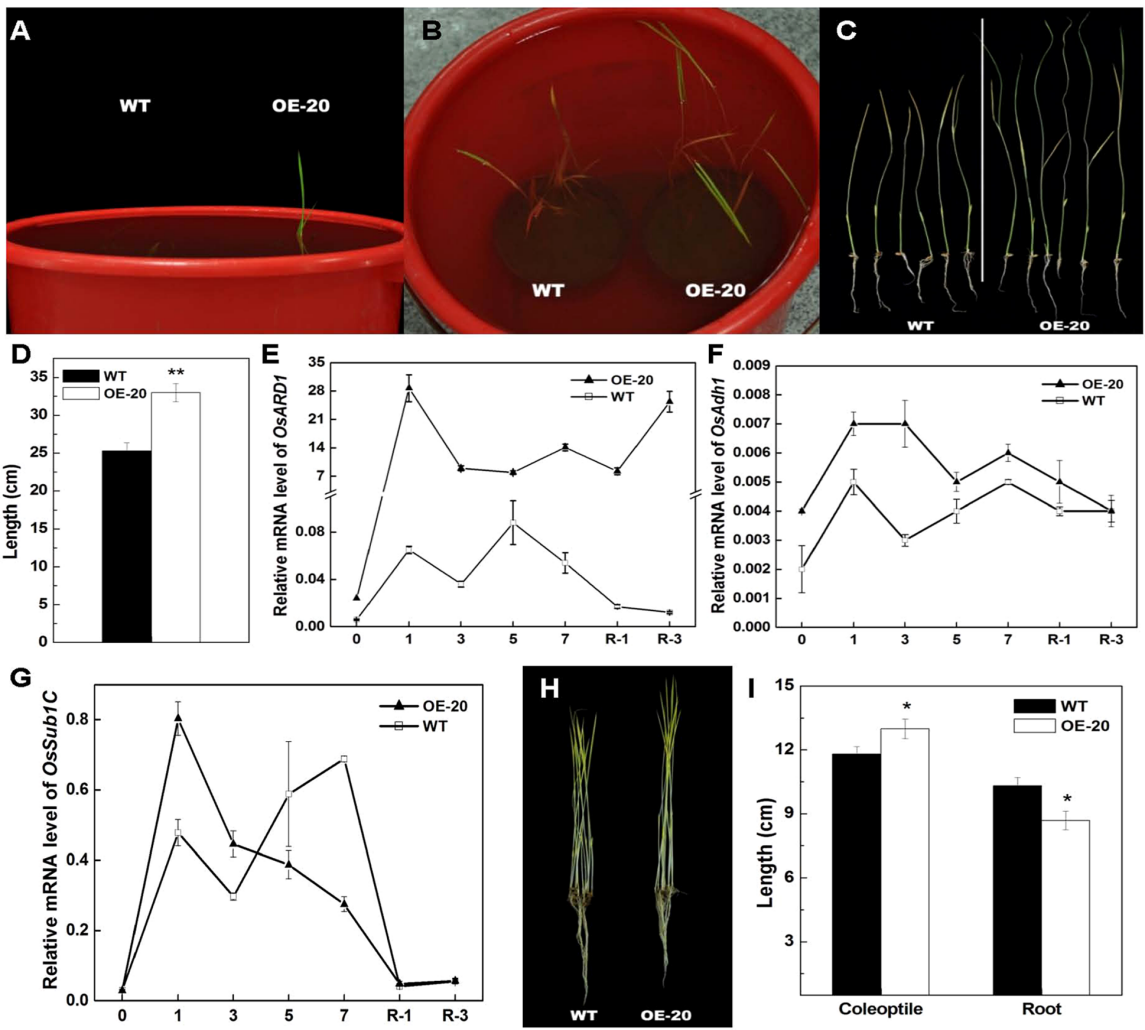
Elongation of shoots of OsARD1-OE and WT seedlings under submergence treatment and the phenotype of dark-grown seedlings. **(A**–**D)** Submergence treatment of WT and OsARD1-OE (OE-20). One-day-old seedling after sowing of WT and OsARD1-OE line were completely submerged under 35 cm of water to grow. The time of leaf from water to air and the length of shoot were recorded. **(A)** and **(B)** The phenotype of WT and OsARD1-OE line at the 7^th^ and 10^th^ day under submergence condition, respectively. **(C)** and **(D)** The shoot length of WT and OsARD1-OE after the submergence 10 days. ** denotes t test at 0.01 significance probability level (n = 10). **(E**–**G)** qRT-PCR analysis of *OsARD1* and submergence-related genes. **(H)** and **(I)** The phenotype of dark-treated seedlings. WT and OsARD1-OE were planted in the dark in climate chamber (28°C). The phenotype and the length of shoot and root were observed and measured after 14 days. **(H)** The phenotype of etiolation seedling of WT and OsARD1-OE line under dark condition. **(I)** The length of shoot and root of WT and OsARD1-OE line. ^*^ denotes t test at 0.05 significance probability level (n = 17).

Ethylene is a major regulator of submergence responses in rice. Since the ethylene release rate was elevated in OsARD1-OE plants, we performed the experiment of submergence stress using OsARD1-OE and WT seedlings. It was observed that the leaves of OsARD1-OE seedlings were out of water after 6 days of treatment, while the leaves of WT seedlings were out of water after 8 days of treatment (**Figures 5A–C**), indicating that the elongation of OsARD1-OE seedlings was faster than WT seedlings under the submergence stress. The length of shoot in OsARD1-OE seedlings was significantly longer than that in WT seedlings, suggesting that overexpression of *OsARD1* promoted the elongation of shoot to overcome submergence stress in rice (**Figures 5A–D**).

We also analyzed the expression of *OsARD1* and submergence-related genes under the submergence stress. The result showed that *OsARD1* expression was rapidly and highly induced in both WT and OsARD1-OE plants under the submergence stress. However, the extent of induced level in OsARD1-OE seedlings was greater than that in WT seedlings, particularly at the beginning of submergence treatment and on the 3 days after restoring (**Figure 5E**). The mRNA level of *alcohol dehydrogenase1* (*Ahd1*), a key gene of ethanol fermentation and promoting the coleoptile elongation under hypoxia, exhibited a stronger induction in OsARD1-OE than that in WT (**Figure 5F**), consistent with the phenotypic results that OsARD1-OE plants had a faster elongation under submergence stress compared with WT plants.

In rice, *Sub1A* is only present in submergence tolerance-specific rice accessions and has an important role in submergence stress (Xu et al., 2006). Zhonghua11 was a submergence-intolerant cultivar without *Sub1A*. Therefore, we analyzed *Sub1C* expression in OsARD1-OE and WT plants under submergence stress and found that the mRNA induction of *Sub1C* was stronger in OsARD1-OE plants than that in WT plants at the beginning of treatment, but *Sub1C* expression was extremely reduced in OsARD1-OE plants and significantly lower than that in WT plants after 3 days of treatment (**Figure 5G**). This result was in agreement with the expression pattern of *Sub1C* in submergence-tolerant rice varieties.

We further tested the classical triple reaction of ethylene in OsARD1-OE plants. In *Arabidopsis*, dark-grown seedlings treated with ethylene exhibit the inhibition of hypocotyls and root elongation (Resnick et al., 2006). However, dark-grown rice seedlings treated with ethylene exhibit the contrary phenotype, which was the promotion of coleoptile elongation and the inhibition of root elongation (Ma et al., 2013). We observed the growth of etiolated seedlings and measured coleoptile and root length of WT and OsARD1-OE lines grown in the dark. The result indicated that coleoptile length of OsARD1-OE seedlings was significantly longer than that of WT seedlings, and root length was significantly shorter than that of WT (**Figures 5H, I**), which was similar to the phenotypes of seedlings treated with ethylene under the darkness. The result confirmed that overexpression of *OsARD1* improved the submergence tolerance in rice seedling through the elevated ethylene.

### Overexpression of *OsARD1* Reduced the Sensitivity to Drought Stress at Seedling Stage

**Figure 6:**
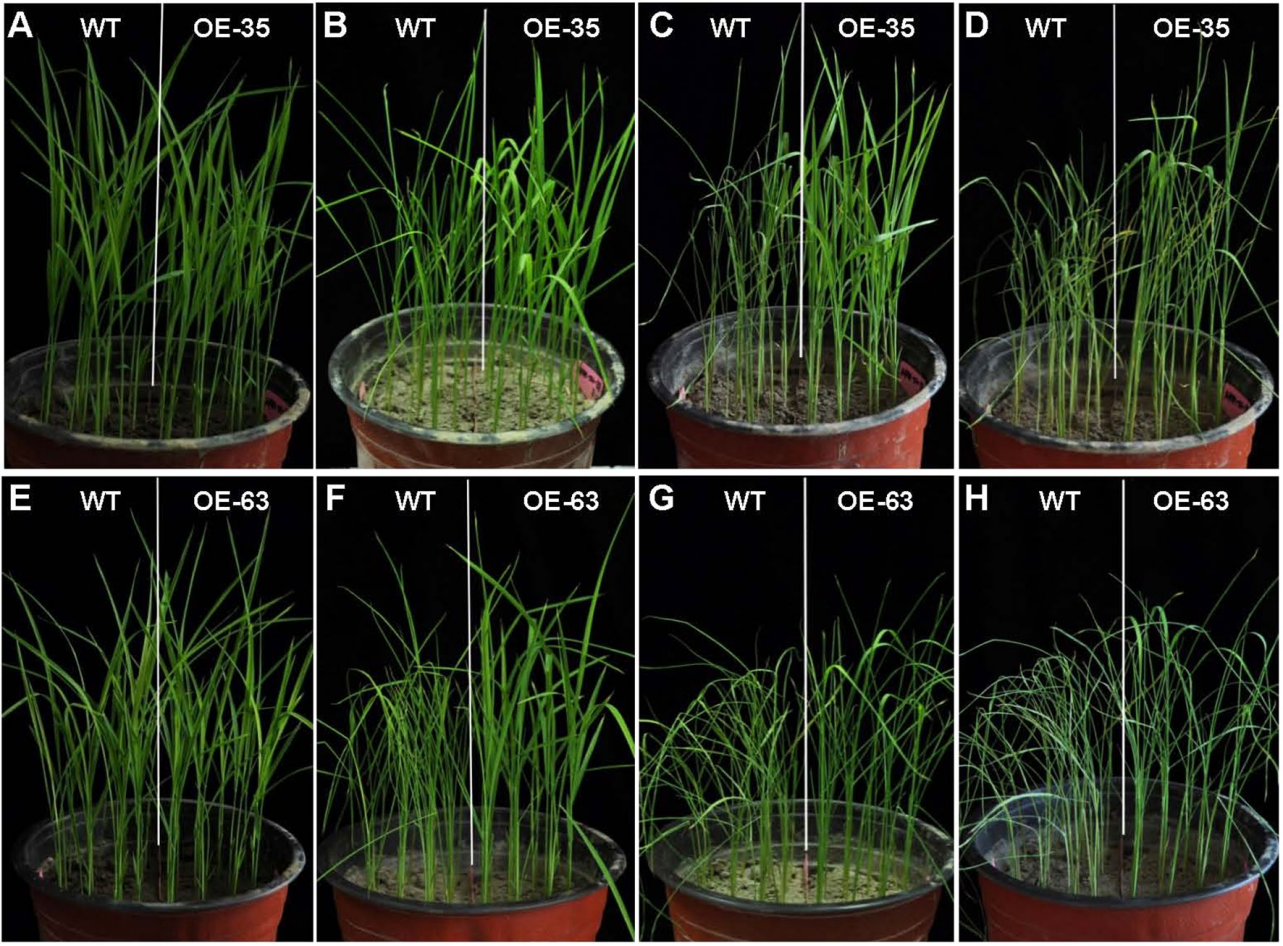
Phenotype of WT and OsARD1-OE seedlings under drought stress. **(A**–**H)** The phenotype of WT and two OsARD1-OE lines (OE35 and OE63) before and after drought treatment. WT and OsARD1-OE plants were planted in the same pot, and 2-week-old seedlings were subject to drought treatment by stopping water. **(A)** and **(E)** Before treatment. **(B)** and **(F)** 7 days after treatment. **(C)** and **(G)** 8 days after treatment. **(D)** and **(H)** 9 days after treatment.

Considering induction expression of *OsARD1* in abiotic stresses, the transgenic seedlings overexpressing *OsARD1* were subject to drought stress. OsARD1-OE and WT plants were planted in the same pot and subject to drought stress. Before the treatment, OsARD1-OE plants from two independent lines were similar to WT plants from the appearance (**Figures 6A, E**). WT leaves began to display dehydrated symptom, while the leaves of OsARD1-OE lines were still normal at the 7^th^ day of drought stress (**Figures 6B, F**). At the 8^th^ day of drought stress, all the leaves of WT rolled up and drooped to a needle-like shape, while OsARD1-OE lines began to appear slight dehydration symptom (**Figures 6C, G**). At the 9^th^ day of treatment, all WT plants were withered and shriveled, while OsARD1-OE lines were still green, and partial leaves were still flat (**Figures 6D, H**). These results showed that overexpression of *OsARD1* reduced the sensitivity and increased the tolerance to drought stress in rice at seedling stage.

### Overexpression of *OsARD1* Reduced the Sensitivity to Osmotic Stress in Rice at Seedling Stage

**Figure 7:**
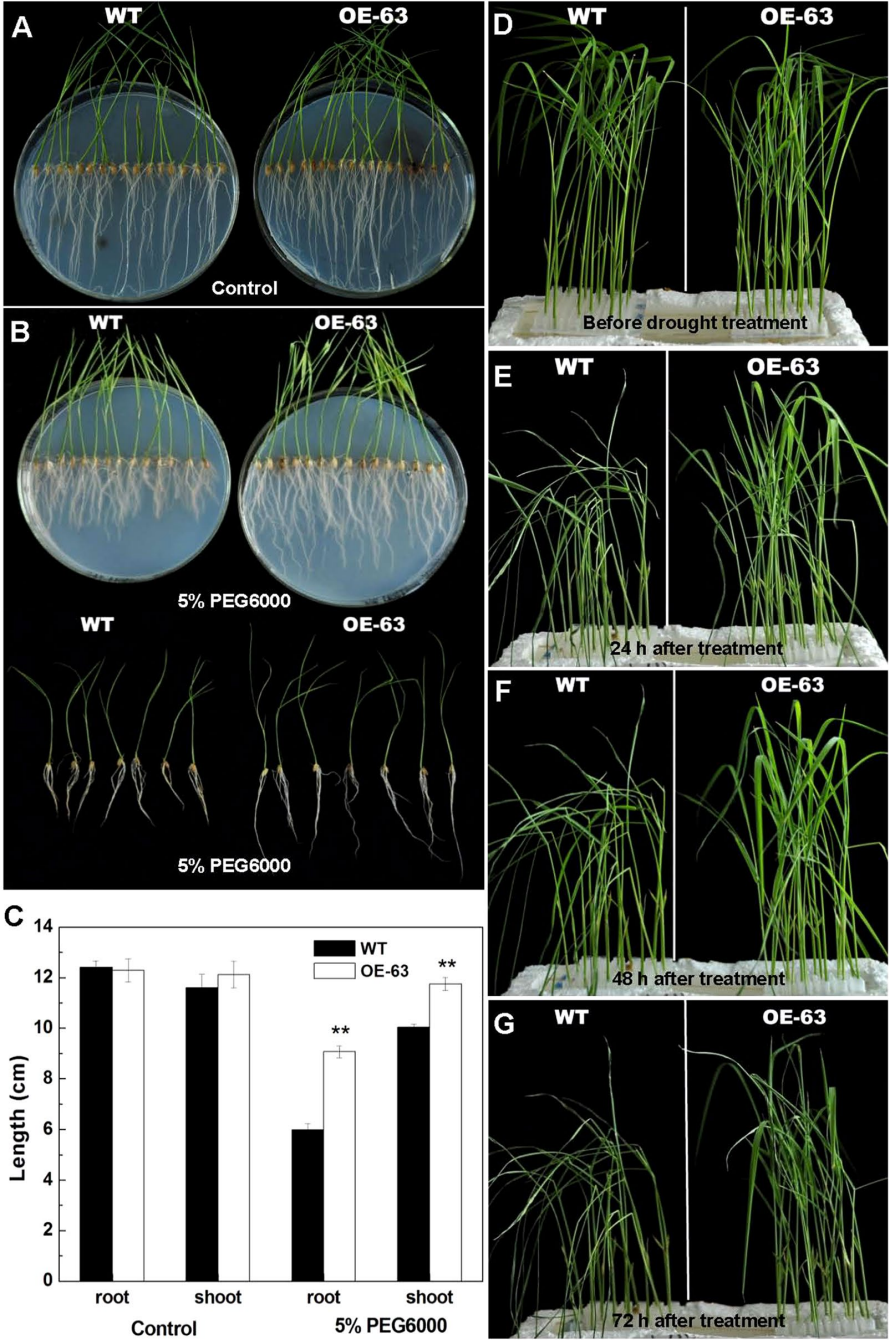
Phenotype of WT and OsARD1-OE seedlings under osmotic stress. **(A**–**C)** Phenotype of WT and OsARD1-OE (OE-63) in control group **(A)** and treatment group **(B)** after 15 days. WT and OsARD1-OE plants were sowed on agar medium without PEG6000 (control group) and agar medium with 5% PEG6000 (treatment group). **(C)** The length of root and shoot of WT and OsARD1-OE plants. ** denotes t test at 0.01 significance probability level (n = 13–14). **(D**–**G)** Phenotype of WT and OsARD1-OE plants in nutrient solution with 20% PEG6000. Two-week-old seedlings of WT and OsARD1-OE in normal nutrient solution were transferred into the nutrient solution with 20% PEG6000 to be subject to the osmotic stress. **(D)** Before treatment. **(E)** Treatment after 24 h. **(F)** Treatment after 48 h. **(G)** Treatment after 72 h.

To further explore the function of *OsARD1* in the response to drought stress, two methods were adopted to treat OsARD1-OE lines using PEG6000 which was frequently used for simulating drought stress. Firstly, we sowed the seeds of WT and OsARD1-OE lines on agar medium with 5% PEG6000 (the treatment group) and agar medium without PEG6000 (the control group). After 15 days, we observed that the length of shoot and root of OsARD1-OE lines was significantly longer than that of WT in the treatment group (**Figures 7B, C**), while no significant difference in the control group (**Figures 7A, C**). In addition, we found that PEG treatment has a stronger impact on root growth than shoot growth (**Figures 7B, C**). The root length of WT plants decreased by 50% in the treatment group compared with the control group, while the root length of OsARD1-OE plants was reduced only about 30% (**Figures 7B, C**). In contrast, the shoot length of WT plants had about 15% reduction in the treatment group compared with the control group, while there was no significant difference in shoot length of OsARD1-OE plants between the control group and the treatment group (**Figures 7B, C**).

We also transferred the 2-week-old seedlings of WT and OsARD1-OE plants from the normal nutrient solution to the nutrient solution containing 20% PEG6000. It was observed that the leaves of WT began to roll up into a needle-like shape after treated for 3 h, while OsARD1-OE plants were same as before treatment. After 24 h of treatment, the leaves of WT became withered and drooped, while OsARD1-OE seedling began to appear slight dehydration symptoms (**Figure 7E**). All the leaves of WT were withered 48 h later, while OsARD1-OE seedling still displayed slight dehydration symptoms (**Figure 7F**). After treatment 72 h, the leaves of WT seedlings became dry or even died, while OsARD1-OE seedlings still had flat leaves (**Figure 7G**). Taken together, the results showed that overexpression of *OsARD1* reduced the sensitivity to osmotic stress and drought in rice at seedling stage, suggesting that *OsARD1* might play a key role in adaptation to drought stress.

### Overexpression of *OsARD1* Reduced the Sensitivity to High Salinity Stress at Seedling Stage

**Figure 8:**
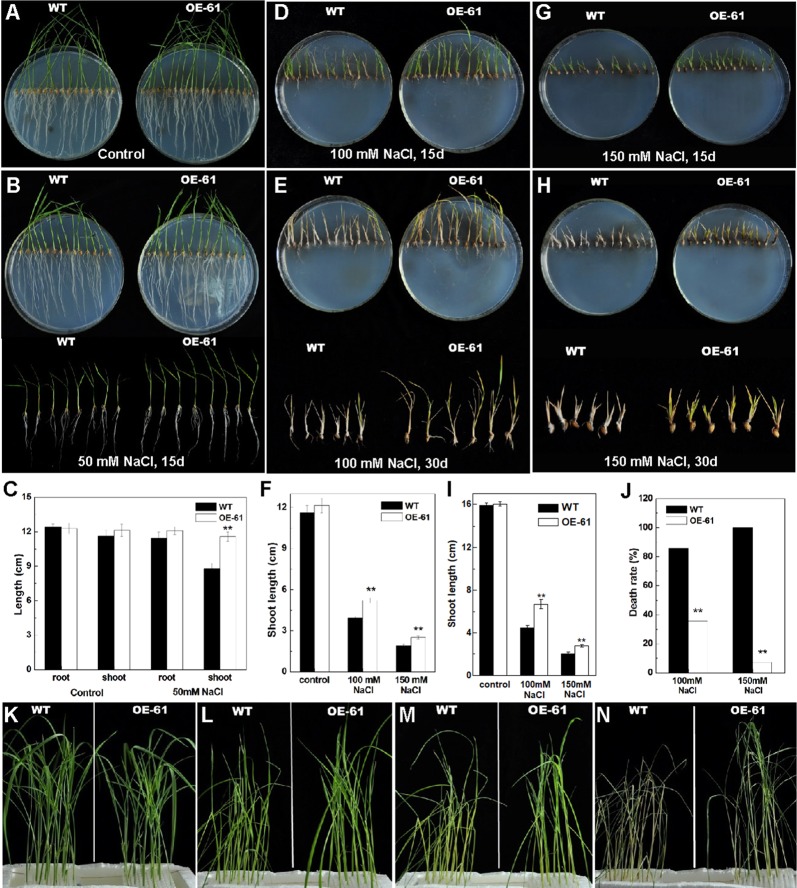
Phenotype of OsARD1-OE and WT seedlings in salinity treatments. **(A**–**J)** Phenotype of OsARD1-OE (OE-61) and WT on agar medium with different salt concentration. The germinated seeds of OsARD1-OE and WT were sowed on agar medium without NaCl (control group) and with different salt concentrations including 50, 100, and 150 mM NaCl (treatment group) for phenotype observation. **(A)** The control group after 15 days. **(B)** The treatment group with 50 mM NaCl after 15 days. **(C)** The length of root and shoot of WT and OsARD1-OE seedlings in control group and 50 mM NaCl treatment group. **(D)** and **(E)** The treatment group with 100 mM NaCl after 15 and 30 days, respectively. **(F)** The shoot length of WT and OsARD1-OE seedlings in the treatments with 100 and 150 mM NaCl after 15 days. **(G)** and **(H)** The treatment group with 150 mM NaCl after 15 and 30 days, respectively. **(I)** Shoot length of WT and OsARD1-OE seedlings in the treatments with 100 and 150 mM NaCl after 30 days. **(J)** Death rate of WT and OsARD1-OE seedlings in 100- and 150-mM NaCl treatment group after treatment of 30 days. **(K**–**N)** Phenotype of WT and OsARD1-OE (OE-61) in nutrient solution with 200 mM NaCl. Two-week-old seedlings of WT and OsARD1-OE in normal nutrient solution were transferred into the nutrient solution with 200 mM NaCl to be subject the salt stress. **(K)** Before treatment. **(L)** Treatment after 24 h. **(M)** Treatment after 48 h. **(N)** Treatment after 72 h. ** denotes t test at 0.01 significance probability level (n = 13–15).

We tested the response of transgenic lines to salinity stress considering *OsARD1* was induced by salt stress. The germinated seeds of WT and OsARD1-OE lines were sowed on agar medium with different salt concentrations including 0 (control), 50, 100, and 150 mM NaCl. We found that there was no significant difference in the length of shoot and root of WT and OsARD1-OE lines in the control group (**Figures 8A, C**), while there was significant difference in the treated group with different salt concentration gradient. In 50-mM NaCl treatment group after 15 days of treatment, the shoot length of OsARD1-OE plants was significantly longer than that of WT, and the root length of OsARD1-OE plants was slightly longer than that of WT (**Figures 8B, C**).

It was observed that higher concentration of salt inhibited the growth of shoot and root, especially the root (**Figures 8D–I**). In 100-mM NaCl treatment group, the shoot length of OsARD1-OE plants was significantly longer than that of WT after 15 and 30 days of treatment, even up to 30% longer after 30 days of treatment (**Figures 8D–F**). However, the root growth was seriously suppressed in both OsARD1-OE and WT plants, and there was no significant difference between them (**Figures 8D, E**). In 150-mM NaCl treatment group, the root growth was completely inhibited. The shoot growth was also seriously inhibited, with the shoot length of OsARD1-OE plants significantly longer than that of WT plants after 15 and 30 days of treatment (**Figures 8G–I**). When WT plants were dry and nearly dead, OsARD1-OE plants were still alive and had green leaves at 30 days after treatment (**Figure 8H**). The mortality of OsARD1-OE plants was significantly lower than that of WT in both 100- and 150-mM NaCl treatment groups (**Figure 8J**), indicating that OsARD1-OE plants were more resistant to salt stress than WT plants.

Two-week-old seedlings of WT and OsARD1-OE lines in normal nutrient solution were transferred into the nutrient solution with 200 mM NaCl (**Figure 8K**). After 24 h of treatment, we observed that the leaves of WT rolled up and drooped, while the leaves of OsARD1-OE was still flat and erect (**Figure 8L**). After another 24 h, WT plants began to turn yellow and dry, while OsARD1-OE plants were still green (**Figure 8M**). After 72 h of treatment, WT were all withered to die, while OsARD1-OE lines were still alive and had green leaves (**Figure 8N**). These results demonstrated that overexpression of *OsARD1* reduced the sensitivity to salt and improved the tolerance to salt in rice.

### Increased Density of Trichome and Stomata on Leaves in *OsARD1*-Overexpressing Plants

**Figure 9:**
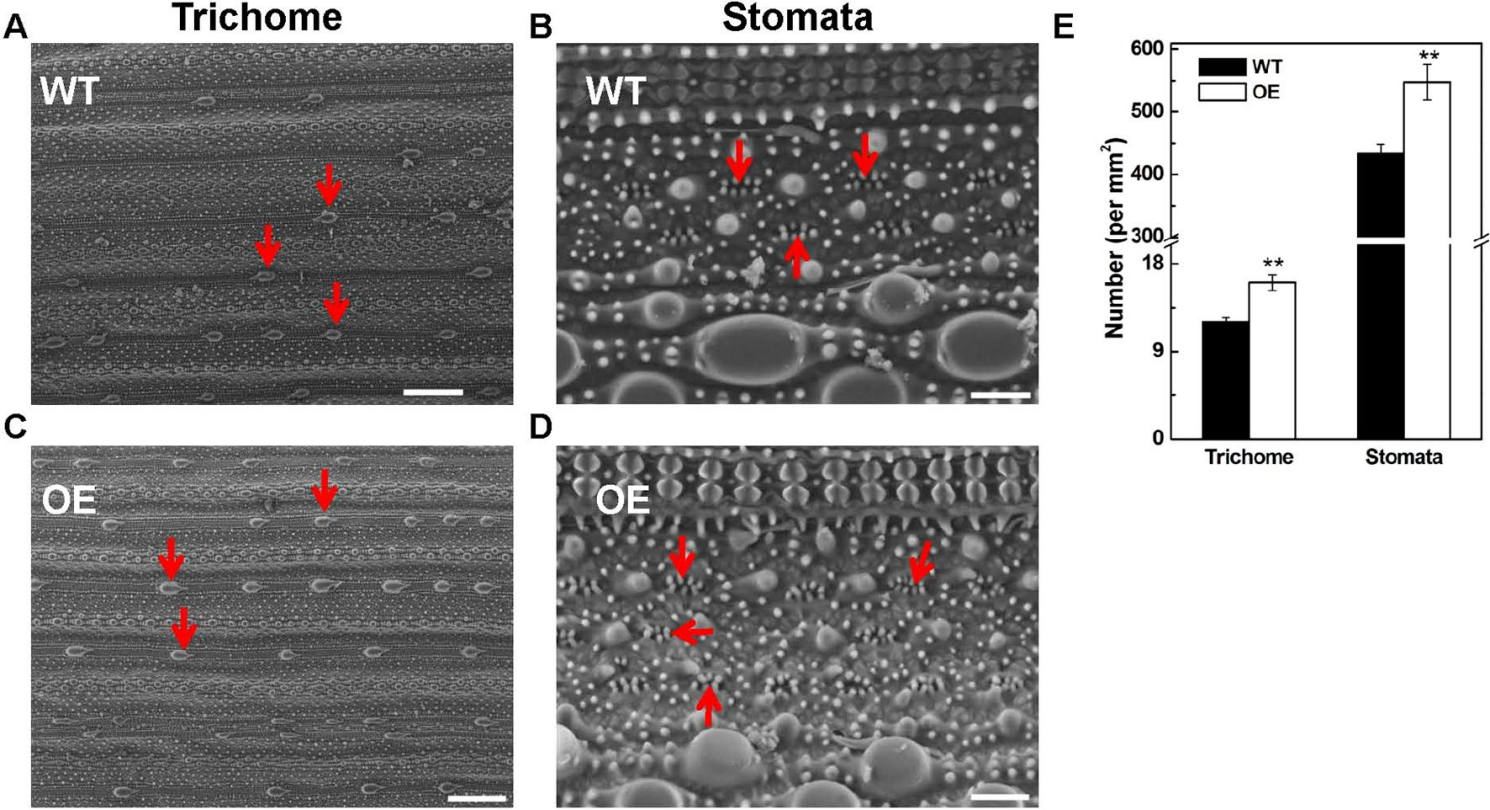
Microscopic observation of leaf surface in WT and OsARD1-OE lines. **(A)** and **(C)** The observation of trichomes (indicated by red arrowheads) in WT and OsARD1-OE (OE-63) leaves. Scale bars: 200 μm. **(B)** and **(D)** The observation of stomata (indicated by red arrowheads) in WT and OsARD1-OE (OE-63) leaves. Scale bars: 20 μm. **(E)** The density of trichomes and stomata in WT and OsARD1-OE (OE-63) line. ** denotes t test at 0.01 significance probability level (n = 5).

The tolerance to abiotic stresses is closely related to trichome and stomata structure of leaves; therefore, we observed the trichome and stomata structure in OsARD1-OE and WT plants using scanning electron microscope (Hitachi, TM3030Plus, Japan). The result showed that the density of trichome and stomata in OsARD1-OE plants increased significantly compared with that in WT plants (**Figures 9A–E**). The number of trichomes and stomata increased by 30 and 25% in OsARD1-OE plants compared with WT plants, respectively (**Figure 9E**). The increased trichomes may facilitate the transgenic plants to adapt to biotic and abiotic stresses by reducing the moisture loss and protect plant from high temperature, mechanical damage, and other environmental damage.

### Enhanced Water Holding Capacity and Water Content in *OsARD1*-Overexpressing Plants

**Figure 10:**
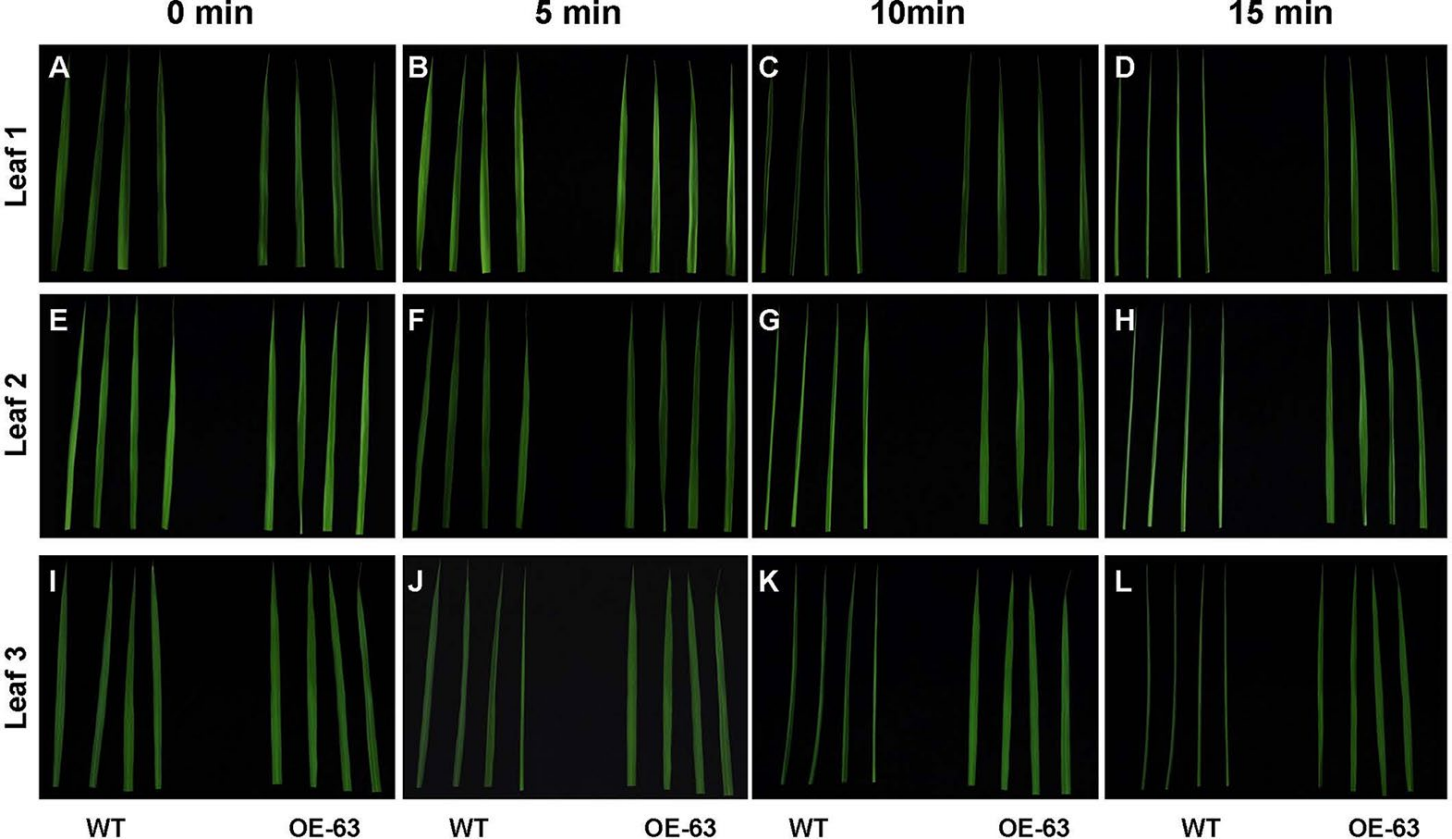
Excised treatment of leaves in WT and OsARD1-OE plants. **(A**–**L)** The phenotype of different leaf-aged leaves in excised treatment. Diffverent leaf-aged leaves including the penultimate, antepenultimate, and the fourth leaves at the bottom were excised from 60-day-old plants of OsARD1-OE (OE-63) and WT to be exposed in air. The phenotype was observed at different times (0, 5, 10, and 15 min). **(A**–**D)** The penultimate leaves (leaf 1). **(E**–**H)** The antepenultimate leaves (leaf 2). **(I**–**L)** The fourth leaves at the bottom (leaf 3).

To study whether overexpression of *OsARD1* improves water holding capacity in rice, we excised leaves at different leaf age including the penultimate, antepenultimate, and the fourth leaves at the bottom from main stem of WT and OsARD1-OE lines to expose in the air (**Figures 10A, E, I**). It was observed that the leaf tip at different leaf age in WT plants began to roll up to the adaxial side at 5 min after excision, while there is no obvious change in OsARD1-OE leaves (**Figures 10B, F, J**). All the different leaves of WT plants showed severe dehydration phenotype after excision 10 min, which the whole leaves rolled up to the adaxial side along the midrib. While the different leaves of OsARD1-OE plants were still same as before the excision (**Figures 10C, G, K**). After excision 15 min, the different leaves of WT plants displayed seriously dehydrated symptoms, which the whole leaves rolled up into a needle-like shape. The leaves of OsARD1-OE plants exhibited slightly dehydrated symptoms with the leaf tips rolled up to the adaxial side (**Figures 10D, H, L**).

We also measured the WC in OsARD1-OE lines and WT. The results indicated that WC and RWC in the four tested transgenic lines were elevated compared with WT plants (**Table 1**). RWC and WC increased about 4–8% and 2–5% in different OsARD1-OE lines, respectively. These results indicated that overexpression of *OsARD1* enhanced the water holding capacity and WC in rice, helping to understand the reduction of sensitivity to drought and salt.

**Table 1:** Relative water content (RWC) and water content (WC) in WT and OsARD1-OE lines.

Line	Mean of RWC (%)	Mean of WC (%)
WT	83.17 ± 3.42	63.74 ± 1.68
OE-20	89.08 ± 1.53 ^n.s^	65.50 ± 0.49 ^n.s^
OE-35	87.53 ± 4.54 ^n.s^	66.21 ± 1.03 ^n.s^
OE-61	89.86 ± 3.37 ^n.s^	66.61 ± 0.75 ^n.s^
OE-63	91.85 ± 5.34 ^n.s^	68.78 ± 0.20 *

The penultimate leaves at heading stage were sampled to measure RWC and WC. Three independent replicates were carried out in WT and OsARD1-OE lines. * denotes t test at 0.05 significance probability level. ^n.s^ represents no significant difference by Student t-test.

### Ethylene Synthesis and Stress-Related Genes Are Upregulated in Transgenic Plants Overexpressing *OsARD1*

**Figure 11:**
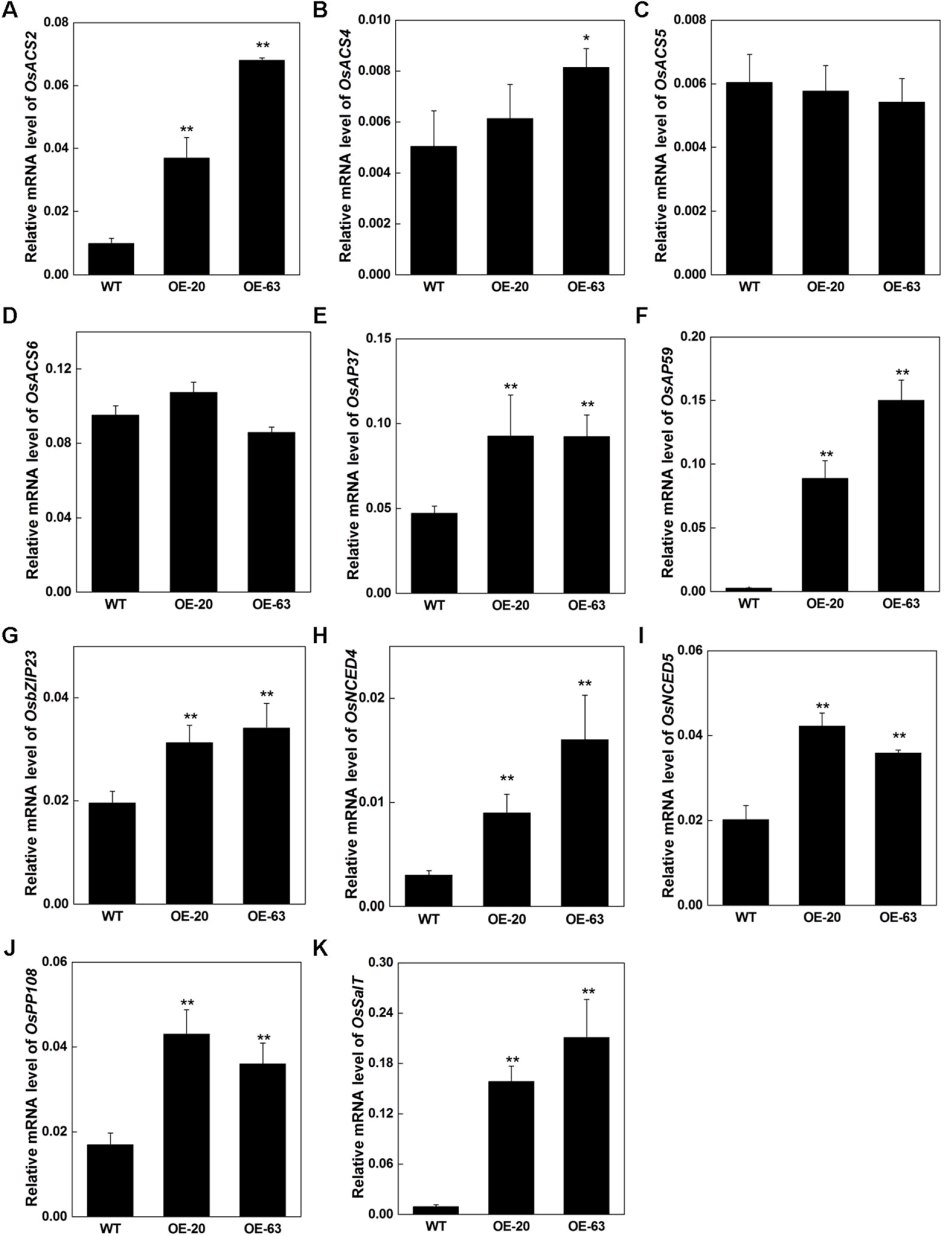
Expression of ethylene synthesis and stress-related genes in OsARD1-OE plants. RNAs were extracted from the 70-day-old leaves of WT and OsARD1-OE plants in T_1_ generation. qRT-PCR was used to analyze the expression of different genes in WT and OsARD1-OE lines. **(A**–**D)** Expression of ethylene synthesis related genes. **(E**–**K)** Expression of stress-related genes. * and ** denote t test at 0.05 and 0.01 significance probability level, respectively (n=5–7).

Due to the elevation of the endogenous ethylene release rate and increase of the tolerance to stresses in OsARD1-OE lines, we further analyzed the expression of several stress-related genes and ethylene synthesis regulatory genes in OsARD1-OE lines. We investigated several members of ACC synthase genes including *OsACS2*, *OsACS4*, *OsACS5*, and *OsACS6*. The result showed that *OsACS2*, whose transcripts can be rapidly induced in mechanical wounding (Lu et al., 2014), was upregulated in OsARD1-OE lines (**Figure 11A**), suggesting that the synthesis of ethylene was enhanced in OsARD1-OE lines. *OsACS4* was slightly upregulated in OsARD1-OE lines (**Figure 11B**), whereas the expression of *OsACS5* and *OsACS6* did not show significant change in OsARD1-OE lines and WT (**Figures 11C, D**). Two ERF members, *AP37* and *AP59*, induced by drought and high-concentration salinity, were upregulated in OsARD1-OE lines (**Figures 11E, F**), which was in agreement with pervious study that *AP37* and *AP59* was upregulated by drought and salinity resulting enhanced tolerance to drought and salt (Oh et al., 2009). *OsbZIP23*, a transcription factor induced by a wide spectrum of stresses, including drought, salt, ABA, and PEG, was upregulated in OsARD1-OE lines (**Figure 11G**), which is consistent with the result of improved tolerance to drought and salinity in transgenic lines overexpressing *OsbZIP23* (Xiang et al., 2008). *OsNCED4* and *OsNCED5*, two key genes of drought tolerance related with ABA (Zong et al., 2016), were upregulated in OsARD1-OE lines (**Figures 11H, I**). *OsPP108 and OsSalT* were also upregulated in OsARD1-OE lines (**Figures 11J, K**), which was in agreement with studies that *OsPP108 and OsSalT* could be induced by drought and salt stresses (Claes et al., 1990; Singh et al., 2015). These results indicated that *OsARD1* was involved in ethylene synthesis pathway and upregulated the expression of stress-related genes to enhance the tolerance to drought, salt, and osmotic stresses.

## Discussion

### *OsARD1* Plays an Important Role in Ethylene Synthesis Pathway

Previous study showed that *OsARD1* encodes an active ARD that can catalyze the formation of 2-keto-4-methylthiobutyrate (KMTB) to further produce methionine (Met) in the Met salvage pathway (Sauter et al., 2005). In ethylene synthesis pathway, Met was catalyzed to produce S-adenosyl-L-methionine (AdoMet), which was further catalyzed to yield the precursor of ethylene, the 1-aminocyclopropane-1-carboxylic acid (ACC), by ACC synthase (ACS) (Yang and Hoffman, 1984; Wang et al., 2002). Met and AdoMet were regenerated and cycled from 5’-methylthioadenosine (MTA) and KMTB to synthesize ethylene in Yang cycle (Yang and Hoffman, 1984; Wang et al., 2002). Therefore, *OsARD1* was located at the upstream of ethylene synthesis pathway. In this study, we provide insights that *OsARD1* is involved in ethylene synthesis and signaling pathway. Our result shows that the endogenous release rate is significantly elevated in OsARD1-OE lines, as high as 41% compared to the WT. The experiment of etiolated seedlings in dark further confirmed the increase of ethylene in OsARD1-OE transgenic plants and displayed the obvious phenotype of ethylene response. In addition, the expression analysis suggested that ACS genes, which were thought to be a rate-limiting step in ethylene synthesis pathway, were significantly upregulated in OsARD1-OE lines, indicating the increase of ethylene synthesis. Moreover, previous study and our result showed that ethylene can induce the expression of *OsARD1*, indicating that *OsARD1* may be involved in the regulation of ethylene synthesis. Based on these results, we conclude that *OsARD1* play a vital role in ethylene synthesis pathway, although *OsARD1* is not thought to be a limited step.

### *OsARD1* Serves an Important Role in Submergence Stress and in Cultivation of Direct Seeding Rice

It has been found that ethylene is a major regulator of submergence response in rice (Xu et al., 2006). Plant photosynthesis, respiration, and energy metabolism were suppressed under submergence condition due to lack of light, oxygen, and carbon dioxide. Plants need to prepare themselves to survive in submergence stress by morphological and physiological alterations, such as elongation of coleoptile, internodes, and leaves or growing slowly to maintain energy. Under submergence stress, ethylene was induced and responded to the stress signal to guide the elongation growth to overcome stress by coordinating the balance of GA and ABA in rice (Fukao and Bailey-Serres, 2008; Rzewuski and Sauter, 2008; Hattori et al., 2009). The submergence experiments clearly demonstrated that the elongation growth of OsARD1-OE transgenic seedlings was faster than the WT, which was due to the elevated ethylene to increase the tolerance. The mRNA level of *Sub1C*, an ERF, increased significantly in OsARD1-OE plants at the beginning of submergence, implying ethylene was involved in the response to submergence. *Adh1* encodes an alcohol dehydrogenase and is essential for fermentative metabolism under hypoxia (Boamfa et al., 2003). Overexpression of *Adh1* enhanced the hypoxia tolerance in *Arabidopsis* (Shiao et al., 2002), and *adh1* mutant inhibited coleoptile elongation in rice (Saika et al., 2006). Our result showed that *Adh1* expression increased significantly in OsARD1-OE plants under submergence conditions. These results were in agreement with the mechanism of deepwater response under submergence stress, indicating that *OsARD1* is associated with ethylene signal and plays an important role in the submergence stress.

Overexpression of *OsARD1* also significantly promoted the elongation of coleoptile under anaerobic germination in the present study. Tolerance of anaerobic conditions during germination is an essential trait for direct-seeded rice cultivation, which has been increasingly practiced among farmers in both rainfed and irrigated ecosystems. The elevated ethylene in the OsARD1-OE transgenic plants promoted the coleoptile elongation under anaerobic germination and the shoot elongation under the submergence, which made *OsARD1* a potential candidate gene for developing new rice varieties that can be used in direct-seeding systems.

### Elevation of Endogenous Ethylene Improves the Tolerance to Abiotic Stresses in Rice Seedling

Although ARD was induced by ethylene in rice and *Arabidopsis*, the biological function of *OsARD1* remains unclear in plants. In this study, we provide the evidences that *OsARD1* plays an important role in responding to submergence, drought, salt, and osmotic stresses in rice. Our result showed that overexpression of *OsARD1* enhanced the water holding capacity and RWC in OsARD1-OE leaves to reduce the sensitivity to drought, salt, and osmotic stresses at seedling stage. The mean values of RWC and WC did not show significant difference between WT and transgenic plants. It is notable that the RWC and WC were measured in leaves from plants under normal growing conditions. The difference between the transgenic and WT lines might be significant under drought stress condition. We speculated that the reduction of the sensitivity to drought, salt, and osmotic stresses resulted from the increase of ethylene yield in OsARD1-OE plants. On the one hand, ethylene can be induced to accelerate the growth and development in different biotic and abiotic stresses, which reduces the damage from stresses through shortening the growth stages. It may be an adaptation mechanism of the different biotic and abiotic stresses (Tao et al., 2015). The expression profile of *OsARD1* demonstrated that mRNA level of *OsARD1* was strongly induced in senescent leaves, indicating that *OsARD1* might play an important role in rice senescence. This result is in agreement with the induction of ethylene in senescent circumstance. On the other hand, the ethylene synthesis and signal transduction–related genes are regulated resulting in the increase of ethylene yield to enhance the tolerance in different biotic and abiotic stresses. For example, the expression of *ACS* genes in ethylene synthesis was affected by different abiotic stresses and further caused the change of ethylene synthesis (Liang et al., 1992; Du et al., 2014; Yu et al., 2016). Ethylene signal transduction–related genes such as *NTHK1*, *OsETOL1*, *MHZ6/OsEIL1*, and *OsEIL2* were also regulated by different abiotic stresses and resulted in the change of ethylene yield to enhance the tolerance (Cao et al., 2007; Lei et al., 2011; Du et al., 2014; Yang et al., 2015). Moreover, several *ERF* genes associated with the regulation of ethylene signal were found to act important roles in drought and salt stresses in plants (Trujillo et al., 2008; Quan et al., 2010; Zhang et al., 2013 and Lee et al., 2016). Based on our results and previous studies, we hypothesized that *OsARD1* might play an important role in abiotic stresses *via* ethylene signal pathway.

### The Probable Interaction Between *OsARD1* and the Transcription Factors

*OsARD1* encodes an active ARD and acts as a key role in the synthesis of ethylene. The subcellular localization result showed that *OsARD1* exhibited strong fluorescence signal in cell nucleus, suggesting that *OsARD1* might bind the transcription factor to form the complex in cell nucleus. Accordingly, we speculated that *OsARD1* might interact with the key transcription factors in the signal transduction of ethylene to perform the biological function. EIN2 and EIN3 are two core transcription factors of ethylene signal transduction in *Arabidopsis*, which regulate the downstream ethylene response–related genes (Chao et al., 1997; Alonso et al., 1999; Guo and Ecker, 2004; Kendrick and Chang, 2008). We obtained OsEIN2 and OsEIN3 homologs in rice and tested the interaction between OsARD1 and two EIN proteins using one hybrid yeast system, respectively. The result showed that OsARD1 does not directly interact with OsEIN2 and OsEIN3 (data not shown). ERF transcription factors are direct targets of EIN3 in *Arabidopsis*, in which EIN3 can bind to the promoter of *ERFs* to regulate the expression of ethylene response–related genes. Several ERFs, such as *SodERF3*, *OsTSRF*, and *OsERF3*, have been revealed to be induced by different abiotic stresses and play important roles in the tolerance to abiotic stresses including drought, salt, and submergence (Trujillo et al., 2008; Quan et al., 2010; Zhang et al., 2013; Lee et al., 2016). These ERF transcription factors can bind to the ethylene-responsive GCC-box which contains core sequence GCCGCC to regulate the expression of the ethylene response–related genes to further respond to abiotic stresses. Therefore, we analyzed the sequence of *OsARD1* promoter region and found that there were three GCC boxes in this region, suggesting that *OsARD1* may be a target of these ERFs in rice. Further studying the interaction between OsARD1 and ERFs will reveal the possible mechanism of *OsARD1* in the tolerance of stresses.

### *OsARD1* May Reduce the Sensitivity to Drought and Salt Stresses at Seedling Stage Through the Crosstalk of ABA and Ethylene Signaling Pathways

Phytohormones play important roles and coordinate various signal transduction pathways when plants responding to abiotic stress. They regulate internal as well as external environmental signals (Kazan, 2015). ABA has been studied extensively and found to play a crucial role in drought and salt stresses. Ethylene, a gaseous signal molecule, also acts as a crucial role in drought and salt stresses (Xu et al., 2006; Zhang et al., 2013; Yang et al., 2015; Lee et al., 2016). We found that overexpressing *OsARD1* in rice could reduce the sensitivity to drought and salinity stresses at seedling stage. And the expression of *OsNCED4*, a key gene in ABA biosynthesis, was upregulated in transgenic lines overexpressing *OsARD1*. *OsbZIP23*, acting as a central regulator in ABA signaling and positively regulating the expression of *OsNCED4*, was also upregulated in transgenic lines overexpressing *OsARD1*. These results indicated that *OsARD1* might play a role in the crosstalk between ABA and ethylene signaling pathway. Overexpression of *TSRF1*, an ERF transcription factor, improves drought tolerance in rice (Quan et al., 2010). Further assay demonstrated that the expression of ABA synthesis gene *OsSDR* was enhanced and resulted in increased ABA sensitivity in transgenic lines overexpressing *TSRF1*, suggesting the crosstalk between ABA and ethylene signaling pathways (Quan et al., 2010). Overexpression of *RAP2.6*, an AP2/ERF transcription factor, conferred hypersensitivity to exogenous ABA, salt, osmotic, and cold stresses in *Arabidopsis*. Expression analysis showed that *AtABI4* mRNA level was elevated in *abi4* loss-of-function mutant with overexpressing *RAP2.6*, indicating that *RAP2.6* in ethylene signaling pathway can crosstalk with *ABI4*-mediated ABA signaling pathway to regulate the response to stresses (Zhu et al., 2010). Ethylene receptor *ETR1* can modulate plant response to drought and salt stresses *via* ABA sensitivity. *etr1-1* mutant was more sensitive to ABA than the WT, leading to the increased sensitivity to osmotic and salt stress (Wang et al., 2008). Another *etr1-7* mutant decreased the sensitivity to ABA, resulting in the increased the tolerance to osmotic and salt stresses (Wang et al., 2008), indicating that ethylene receptor *ETR1* can crosstalk with ABA to regulate drought and salt tolerance. Based on our results and previous studies, *OsARD1* might reduce the sensitivity to drought and salt stresses at seedling stage through the crosstalk with ABA and ethylene signaling pathways.

## Author Contributions

WL designed the research project. SL, WX, Y-JJ, CY, and WL designed and performed all the experiments and analyzed the data. XX, SZ and BY assisted in the production of transgenic plants, the field experiments and the analysis of the data. WX, W-JL and SL drafted the manuscript. GY, SC and XX helped modifying the manuscript. All authors read and approved the final manuscript.

## Funding

This research was supported by the Tianjin Natural Science Foundation of China (No. 16JCZDJC33400, 17JCYBJC30000 and17JCYBJC41300), the National Natural Science Foundation of China (No.31770343 and 31171515), Tianjin Rice Industrial Technology System of China (No. ITTRRS2018006) and the Tianjin Normal University Doctoral Foundation (No. 043-135202XB1611 and No. 043-135202XB1612)

## Conflict of Interest Statement

The authors declare that the research was conducted in the absence of any commercial or financial relationships that could be construed as a potential conflict of interest.
