# Supplementary figures and images for: Overexpression of OsARD1 Improves Submergence, Drought, and Salt Tolerances of Seedling Through the Enhancement of Ethylene Synthesis in Rice
Source: Front Plant Sci. 2019 Sep 10;10:1088. doi: 10.3389/fpls.2019.01088 (PMC6746970; doi:10.3389/fpls.2019.01088)

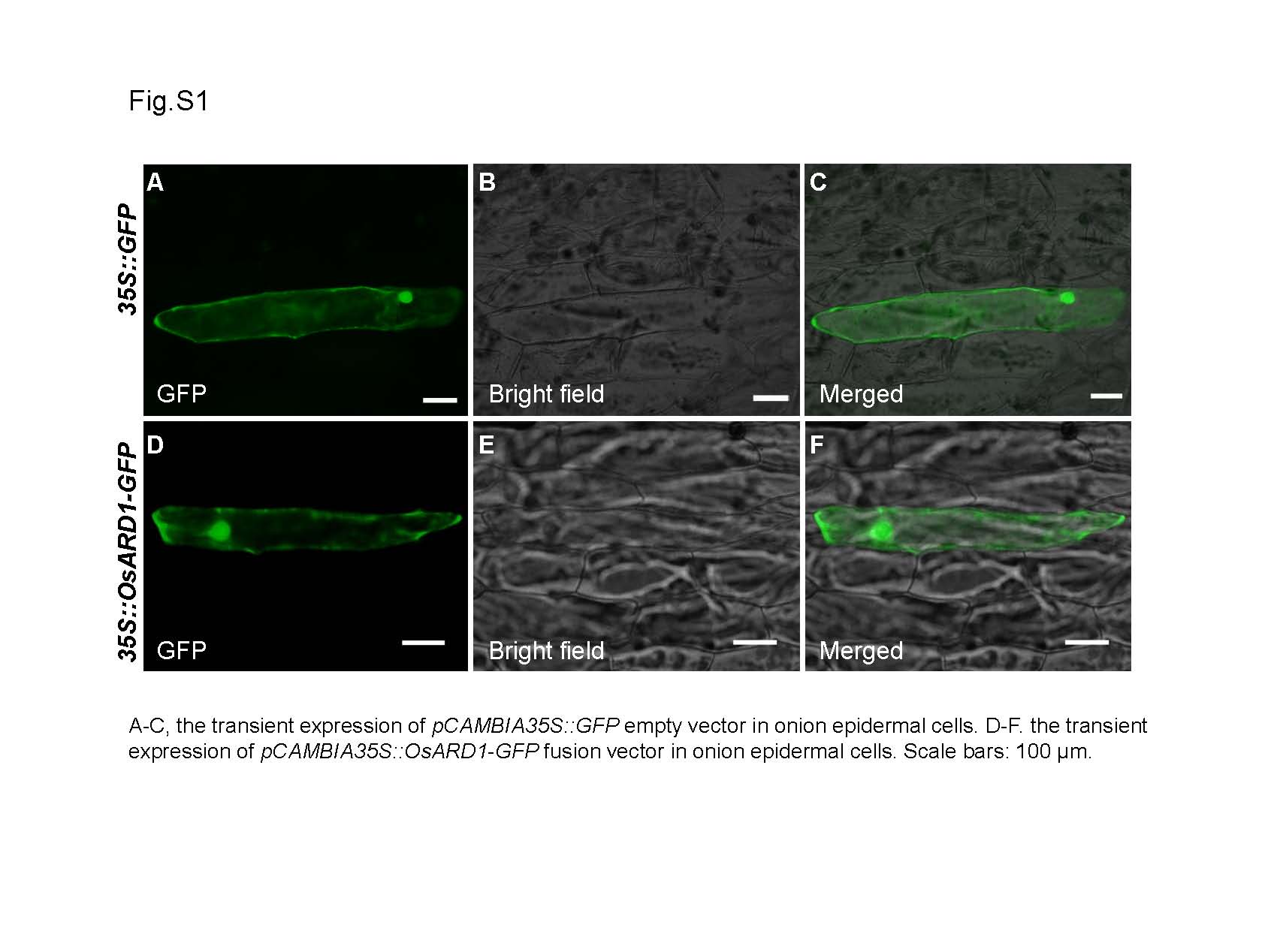

Supplement: Supplementary file 1 [file Image_1.jpg]
